# The HELLP syndrome: Clinical issues and management. A Review

**DOI:** 10.1186/1471-2393-9-8

**Published:** 2009-02-26

**Authors:** Kjell Haram, Einar Svendsen, Ulrich Abildgaard

**Affiliations:** 1 Department of Obstetrics and Gynaecology, Haukeland University Hospital, Bergen, Norway; 2 Department of Pathology, The Gade Institute, Haukeland University Hospital, University of Bergen, Norway; 3 Department of Haematology, Aker University Hospital, N-0514 Oslo, Norway

## Abstract

**Background:**

The HELLP syndrome is a serious complication in pregnancy characterized by haemolysis, elevated liver enzymes and low platelet count occurring in 0.5 to 0.9% of all pregnancies and in 10–20% of cases with severe preeclampsia. The present review highlights occurrence, diagnosis, complications, surveillance, corticosteroid treatment, mode of delivery and risk of recurrence.

**Methods:**

Clinical reports and reviews published between 2000 and 2008 were screened using Pub Med and Cochrane databases.

**Results and conclusion:**

About 70% of the cases develop before delivery, the majority between the 27th and 37th gestational weeks; the remainder within 48 hours after delivery. The HELLP syndrome may be complete or incomplete. In the Tennessee Classification System diagnostic criteria for HELLP are haemolysis with increased LDH (> 600 U/L), AST (≥ 70 U/L), and platelets < 100·10^9^/L. The Mississippi Triple-class HELLP System further classifies the disorder by the nadir platelet counts. The syndrome is a progressive condition and serious complications are frequent. Conservative treatment (≥ 48 hours) is controversial but may be considered in selected cases < 34 weeks' gestation. Delivery is indicated if the HELLP syndrome occurs after the 34th gestational week or the foetal and/or maternal conditions deteriorate. Vaginal delivery is preferable. If the cervix is unfavourable, it is reasonable to induce cervical ripening and then labour. In gestational ages between 24 and 34 weeks most authors prefer a single course of corticosteroid therapy for foetal lung maturation, either 2 doses of 12 mg betamethasone 24 hours apart or 6 mg or dexamethasone 12 hours apart before delivery. Standard corticosteroid treatment is, however, of uncertain clinical value in the maternal HELLP syndrome. High-dose treatment and repeated doses should be avoided for fear of long-term adverse effects on the foetal brain. Before 34 weeks' gestation, delivery should be performed if the maternal condition worsens or signs of intrauterine foetal distress occur. Blood pressure should be kept below 155/105 mmHg. Close surveillance of the mother should be continued for at least 48 hours after delivery.

## Background

It has been known for a long time that preeclampsia may be associated with haemolysis, elevated liver enzymes and thrombocytopenia [1]. Weinstein regarded signs and symptoms to constitute an entity separated from severe preeclampsia and in 1982 named the condition HELLP (H = Haemolysis, EL = Elevated Liver enzymes, LP = Low Platelets) syndrome [2]. The HELLP is currently regarded as a variant of severe preeclampsia or a complication [3-9].

Diagnosis of the complete form of the HELLP syndrome requires the presence of all 3 major components, while partial or incomplete HELLP syndrome consists of only 1 or 2 elements of the triad (H or EL or LP) [3,7,8,10].

The HELLP syndrome, a serious condition in its complete form, is associated with substantial risk for the mother and her foetus [3-6,11-14]. A wide range of complications may arise and the condition represents diagnostic and therapeutic problems; timing and method of delivery are important.

The aim of this review is to present an update on maternal clinical issues of the syndrome, with special focus on diagnosis, complications, surveillance, timing and mode of delivery and risk of recurrence. Perinatal mortality and morbidity are briefly outlined and controversial aspects of corticosteroid (CS) treatment are particularly discussed.

## Methods

A systematic literature search for clinical reports and reviews published between 2000 and 2008 was undertaken using Pub Med and the Cochrane databases. Search words were "HELLP syndrome", and "HELLP syndrome" combined with "diagnosis", respective "clinical symptoms", "complications", "morbidity", "mortality", "management", "treatment", "corticosteroid", "prognosis", "delivery", "post-partum" and "recurrence". Publications were selected for review based on original research, highly regarded earlier publications and comprehensive reviews. The abstracts were read and those publications considered to be relevant were used at the authors' discretion. Some publications have also been found in the reference list in previous publications.

### Occurrence and clinical symptoms

The HELLP syndrome occurs in about 0.5 to 0.9% of all pregnancies and in 10 to 20% of cases with severe preeclampsia [15,16]. In about 70% of the cases, the HELLP syndrome develops before delivery [14] with a peak frequency between the 27th and 37th gestational weeks; 10% occur before the 27th week, and 20% beyond the 37th gestational week [6]. The mean age of pregnant women with HELLP syndrome is usually higher than in women with preeclampsia [3,17]. Most white women with HELLP are multiparous [10]. In the post-partum period the HELLP syndrome usually develops within the first 48 hours in women who have had proteinuria and hypertension prior to delivery [14]. Although variable, the onset of the HELLP syndrome is usually rapid [7]. The majority of women with the HELLP syndrome have had hypertension and proteinuria, which may be absent in 10–20% of the cases [9]. Excessive weight gain and generalized oedema precede the syndrome in more than 50% of the cases [8].

Typical clinical symptoms are right upper abdominal quadrant or epigastric pain, nausea and vomiting. The upper abdominal pain may be fluctuating, colic-like [9,18]. Many patients report a history of malaise some days before presentation [9]. Up to 30–60% of women have headache; about 20% visual symptoms [9]. However, women with a HELLP syndrome might also have unspecific symptoms or subtle signs of preeclampsia or non-specific viral syndrome-like symptoms [9]. The symptoms usually continuously progress and their intensity often changes spontaneously. The HELLP syndrome is characterized by exacerbation during the night and recovery during the day [19].

Women with partial HELLP syndrome have fewer symptoms and develop less complications than those with the complete form [3]. However, a partial or incomplete HELLP syndrome may develop to a complete form of the disorder [7]. Partial or total reversal of the syndrome may also occasionally occur, albeit rarely [18,20].

### The triad signs of haemolysis, elevated liver enzymes and thrombocytopenia

Haemolysis, one of the major characteristics of the disorder, is due to a microangiopathic haemolytic anaemia (MAHA). Red cell fragmentation caused by high-velocity passage through damaged endothelium appears to represent the extent of small vessel involvement with intima damage, endothelial dysfunction and fibrin deposition. Presence of fragmented (schizocytes) or contracted red cells with spicula (Burr cells) in the peripheral blood smear reflects the haemolytic process and strongly suggests the development of MAHA [6,21]. Polychromatic red cells are also seen in blood smears, and increased reticulocyte counts reflect the compensatory release of immature red cells into peripheral blood. Destruction of red blood cells by haemolysis causes increased serum lactate dehydrogenase (LDH) levels and decreased haemoglobin concentrations [22,23]. Haemoglobinaemia or haemoglobinuria is macroscopically recognizable in about 10% of the women [24]. Liberated haemoglobin is converted to unconjugated bilirubin in the spleen or may be bound in the plasma by haptoglobin. The haemoglobin-haptoglobin complex is cleared quickly by the liver, leading to low or undetectable haptoglobin levels in the blood, even with moderate haemolysis [22,23]. Low haptoglobin concentration (< 1 g/L – < 0.4 g/L) can be used to diagnose haemolysis [23-26] and is the preferred marker of haemolysis [27]. Thus, the diagnosis of haemolysis is supported by high LDH concentration and the presence of unconjugated bilirubin, but the demonstration of low or undetectable haptoglobin concentration is a more specific indicator.

Elevation of liver enzymes may reflect the haemolytic process as well as liver involvement. Haemolysis contributes substantially to the elevated levels of LDH, whereas enhanced asparate aminotransferase (AST) and alanine aminotransferase (ALAT) levels are mostly due to liver injury. Plasma glutathione S-transferase-a1 (α-GST or GST-a1) may provide a more sensitive indicator for acute liver damage than AST and ALAT, and allow earlier recognition [28]. However, measurement of α-GST is not widely available, and has not yet found its place in the routine diagnostic procedure [24].

Thrombocytopenia (platelets (PLTs) < 150·10^9^/L) in pregnancy may be caused by gestational thrombocytopenia (GT) (59%), immune thrombocytopenic purpura (ITP) (11%), preeclampsia (10%), and the HELLP syndrome (12%)[29]. PLTs < 100·10^9^/L are relatively rare in preeclampsia and gestational thrombocytopenia, frequent in ITP and obligatory in the HELLP syndrome (according to the Sibai definition). Decreased PLT count in the HELLP syndrome is due to their increased consumption. Platelets are activated, and adhere to damaged vascular endothelial cells, resulting in increased platelet turnover with shorter lifespan [21,30,31].

### Diagnostic criteria

At present, there are two major definitions for diagnosing the HELLP syndrome. In the Tennessee Classification System, Sibai has proposed strict criteria for "true" or "complete" HELLP syndrome (Table 1) [8,9]. Intravascular haemolysis is diagnosed by abnormal peripheral blood smear, increased serum bilirubin (≥ 20.5 μmol/L or ≥ 1.2 mg/100 mL) and elevated LDH levels (> 600 units/L (U/L) [8,32].

**Table 1:** Main diagnostic criteria of the HELLP syndrome

HELLP class	Tennessee Classification	Mississippi classification
1	Platelets ≤ 100·10^9^/L	Platelets ≤ 50·10^9^/L
	AST ≥ 70 IU/L	AST or ALT ≥ 70 IU/L
	LDH ≥ 600 IU/L	LDH ≥ 600 IU/L

2		Platelets ≤ 100·10^9^/L ≥ 50·10^9^/L
		AST or ALT ≥ 70 IU/L
		LDH ≥ 600 IU/L

3		Platelets ≤ 150·10^9^/L ≥ 100·10^9^/L
		AST or ALT ≥ 40 IU/L
		LDH ≥ 600 IU/L

In The Mississippi-Triple Class System, a further classification of the disorder is based on the nadir PLT count any time during the course of the disease (Table 1) [7]. Class 1 and class 2 are associated with haemolysis (LDH > 600 U/L) and elevated AST (≥ 70 U/L) concentration, while class 3 requires only LDH > 600 U/L and AST ≥ 40 U/L in addition to the specific PLT count [7,33,34]. Class 3 HELLP syndrome is considered as a clinical significant transition stage or a phase of the HELLP syndrome which has the ability of progression [34].

The diagnosis of the HELLP syndromes has often been based on different criteria [9]. The condition can be diagnosed simply on biochemical evidence [4,9,14,35-37]. Some authors require the presence of severe preeclampsia together with biochemical substantiation to diagnose HELLP [5,38-42]. Others deal with the HELLP syndrome as partial or incomplete HELLP [43,44]. A number of studies have included women with lack of suspicion or evidence of haemolysis. The syndrome ELLP has been described where there has been no haemolysis [15,45]. The use of different definitions makes comparison of published data difficult [9]. According to Smulian *et al*. the threshold of normal LDH values may be much lower than 600 U/L depending on the laboratory method adopted [41]. Visser and Wallenburg used ALAT > 30 U/L to define abnormality (2 SD above mean in hospital) [20]. Clearly, the analytical method used is important for the diagnostic reference range.

### Differential diagnosis

The HELLP syndrome may be misdiagnosed as viral hepatitis, cholangitis and other acute diseases (Table 2) [6,46]. Other less common, but serious conditions that may mimic HELLP, include ITP, acute fatty liver of pregnancy (AFLP), haemolytic uremic syndrome (HUS), thrombotic thrombocytopenic purpura (TTP) and systemic lupus erythematosus (SLE) [21,32]. These conditions are associated with high maternal mortality and may cause long-term squeals [32]. They may be mistaken for a HELLP syndrome and a careful diagnostic evaluation is required as their therapy is quite different.

**Table 2:** Differential diagnosis of the HELLP syndrome.

1. Diseases related to pregnancy
Benign thrombocytopenia of pregnancy
Acute fatty liver of pregnancy (AFLP)

2. Infectious and inflammatory diseases, not specifically related to pregnancy:
Virus hepatitis
Cholangitis
Cholecystitis
Upper urinary tract infection
Gastritis
Gastric ulcer
Acute pancreatitis

3. Thrombocytopenia
Immunologic thrombocytopenia (ITP)
Folate deficiency
Systemic lupus erythematosus (SLE)
Antiphospholipid syndrome (APS)

4. Rare diseases that may mimic HELLP syndrome
Thrombotic thrombocytopenic purpura (TTP)
Haemolytic uremic syndrome (HUS)

Clinical signs of AFLP vary and there is significant overlap in clinical and biochemical features with the HELLP syndrome [47]. AFLP typically occurs between the 30th and 38th gestational weeks with a 1 to 2 week history of malaise, anorexia, nausea, vomiting, mid epigastric or right upper abdominal pain, headache and jaundice. Hypertension and proteinuria are usually absent. Further examination reveals haemoconcentration, metabolic acidosis, acute liver failure and low grade disseminated intravascular coagulation (DIC) with normal or moderately subnormal PLT count, prolonged prothrombin time (PT) and partial thromboplastin time (PTT), low serum fibrinogen and antithrombin concentrations [32,47,48]. Abnormal blood tests also include leucocytosis, increased levels of creatinine, uric acid, ammonium and liver enzymes such as alkaline phosphatase, AST, ALAT and bilirubin [32,49]. Hypoglycemia and prolongation of prothrombin time may distinguish AFLP from the HELLP syndrome [47]. Ultrasound examination of the liver may reveal increased echogenicity in severe cases of AFLP. Computerized tomography (CT) can well show decreased or diffuse attenuation in the liver. Liver biopsy is recommended as the standard procedure to confirm the diagnosis, but requires an acceptable haemostatic function [32]. Gastrointestinal bleeding, acute renal failure and pancreatitis may complicate AFLP. Most women improve in the course of 1 to 4 weeks post-partum, but AFLP may recur in next pregnancy [47].

ITP is a clinical syndrome with thrombocytopenia which may be manifested as a bleeding disorder with purpura and petechiae. Pregnancy does not increase the incidence of ITP, nor does it exacerbate a preexisting disease. Even with a very low platelet count there is in most cases neither maternal nor fetal morbidity or mortality [29,50,51].

HUS and TTP are thrombotic microangiopathies which share some of the pathophysiological characteristics of the HELLP syndrome including endothelial injury, platelet aggregation, microthrombi, thrombocytopenia and anaemia [49]. Abnormal blood smear, increased LDH and creatinine levels may help in the differentiation. The microvascular injury in HUS affects mainly the kidneys [32]. HUS develops usually in the post-partum period, with signs and symptoms of renal failure [9]. However, most cases arise in children and adolescents caused by a specific enterotoxin produced by Escherichia coli O157:H7. Rare forms may be due to a genetic abnormality in the complement system [52]. TTP, which is an extremely rare condition during pregnancy, is characterized by neurological dysfunction, fever, abdominal pain and bleeding. The spectrum of neurological abnormalities spans from headache to visual disturbances, confusion, aphasia, transient paresis, weakness and seizures. High levels of high-molecular weight von Willebrand factor in maternal serum reflect the virtual absence of the metalloprotease ADAMTS13 enzyme which is required to control the level of the factor [32,53]. Specific tests for this hereditary condition are not readily available in routine clinical laboratories [32]. The mortality of HUS and TTP has decreased due to the use of plasma exchange and intensive care [52].

SLE is an autoimmune disorder characterized by deposits of antigen-antibody complexes in capillaries, with mild to severe clinical findings. SLE may affect multiple organ systems (kidneys, lungs, heart, liver and brain). The clinical and laboratory findings in women with lupus nephritis are similar to those with severe preeclampsia. Antiphospholipid antibodies (lupus anticoagulant and/or anticardiolipin antibodies) are present in 30–40% of the cases, while thrombocytopenia occurs in 40–50% and haemolytic anaemia in 14–23% of the women with SLE. Cerebral lesions and symptoms may develop because of vasculitis and/or cerebro-vascular occlusion that might lead to seizures [32]. In the so-called antiphospholipid syndrome (APS) antiphospholipid antibodies are associated with recurrent thrombosis (in arteries and veins) and pregnancy loss. APS may also occur as a primary disease, unrelated to SLE. Development of HELLP syndrome in women with an established APS syndrome may be more frequent than previously thought [54].

Folate deficiency is common in pregnancy, but its progression to megaloblastosis is rare. Haemolytic anaemia, thrombocytopenia, and coagulopathy due to folate deficiency may mimic the incomplete HELLP syndrome [55].

### Complications of the HELLP syndrome

The HELLP syndrome is associated with both maternal and neonatal complications. Frequencies of reported serious complications are summarized in table 3[56-77].

**Table 3:** Complications reported in the HELLP syndrome

**Maternal complications**	**Occurrence (%)**	**References**
Eclampsia	4–9	[56]
*Abruptio placentae*	9–20	[3,9,35,45,57]
DIC	5–56^1^	[35,36,58]
Acute renal failure	7–36	[4,57,59,60]
Severe ascites	4–11	[36]
Cerebral oedema	1–8	[14,36]
Pulmonary oedema	3–10	[3,36,60]
Wound hematoma/infection^2^	7–14	[3,56]
		
Subcapsular liver hematoma	Between 0.9% and <2%	[9,14,61-64]
Liver rupture	>200 cases or about 1.8%	[45,65]
Hepatic infarction	>30 cases combined with APS	[66]
Recurrent thrombosis	Associated with prothrombin gene 20210a mutation	[67]
		
Retinal detachment	1	[14]
Cerebral infarction	Few case reports	[68-71]
Cerebral Haemorrhage	1.5–40^3^	[3,36,70]
Maternal death	1–25	[5,11]
**Foetal/neonatal complications**		
Perinatal death	7.4–34	[9,72,73].
IUGR	38–61	[5,72,74]
Preterm delivery^4^	70 (15% < 28 gestational weeks)	[9]
Neonatal thrombocytopenia ^5^	15–50	[5,75]
RDS	5.7–40	[72,76,77]

^1 ^depending on diagnostic criteria

^2 ^after Caesarean section

^3 ^in a highly selected group of patients

^4 ^associated with increased risks of RDS, IVH and CP

^5^PLTs < 100·10^9^/L

Laboratory thresholds that indicate more than 75% risk of serious maternal morbidity are LDH concentration > 1400 U/L, AST > 150 U/L, ALAT > 100 U/L, and uric acid concentration >7.8 mg/100 ml (> 460 μmol/L)[6]. Interestingly, clinical symptoms, such as headache, visual changes, epigastric pain and nausea-vomiting, have been suggested to be better predictors of adverse maternal outcome than laboratory parameters [57].

Spontaneous rupture of a subcapsular liver haematoma in pregnancy is a rare, but life threatening complication that occurs 1 in 40,000 to 1 in 250,000 deliveries [63] and about 1% to < 2% of the cases with the HELLP syndrome. Rupture most often occurs in the right liver lobe [9,14,61-64]. The symptoms are sudden-onset severe pain in the epigastric and right upper abdominal quadrant radiating to the back, right shoulder pain, anaemia and hypotension. The condition may be diagnosed by ultrasound, CT or magnetic resonance imaging (MRI) examination [61-63,78]. Hepatic rupture may also occur in the post-partum period [79]. A few cases of liver infarctions associated with antiphospholipid syndrome and HELLP syndrome have been reported [66]. Even recurrent deep vein thromboses and palmar skin lesions have been reported in a woman with prothrombin gene 20210a mutation and antiphospholipid antibodies complicated by the HELLP syndrome [67].

More common and serious maternal complications are *abruptio placentae*, DIC and subsequent severe post-partum bleeding (Table 3) [5]. Bilateral permanent visual loss associated with retinopathy (Pursher-like) is a rare ophthalmic complication during pregnancy [80]. In the literature there are several case reports on cerebral bleeding associated with the HELLP syndrome [68-71]. In a report by Sibai *et al*. on the outcome of 442 pregnancies complicated by HELLP cerebral bleeding was not mentioned as a complication [14]. Audibert *et al*. report cerebral bleeding to occur in 1.5% of the cases [3]. Contrary to this, in a highly selected group of 37 women with the HELLP syndrome that was transferred to an obstetric intensive care unit in Turkey, 15 women (40%) had cerebral haemorrhage. In this study CT and MRI were used as diagnostic tools [36]. The risk of stroke is not increased during pregnancy itself. However, the risk of cerebral infarction and intracerebral haemorrhage is increased some weeks after delivery [81]. This is reflected by some case reports of cerebral infarction after delivery as a complication to the HELLP syndrome [68-71]. Life-threatening neurological complications of the HELLP syndrome are rare, but incorporate large cerebral or brain stem haemorrhage, thrombosis and infarctions or cerebral oedema complicated by brain herniation [6]. Wound haematoma and infection are frequent phenomena in women with the HELLP syndrome undergoing Caesarean section [82].

### DIC

Activation of vascular endothelium and of platelets, haemolysis and liver damage are the basic pathophysiological features characteristic for the HELLP syndrome, each predisposing to DIC [83,84]. In a retrospective cohort study, 38% of pregnant women with the HELLP syndrome developed DIC (PLTs < 100·10^9^/L, low serum fibrinogen concentration (< 3 g/L), fibrin degradation products (FDP) (>40 μg/ml = 40 mg/L) most often related to placental abruption [45]. Low antithrombin concentration may be caused by liver dysfunction with decreased synthesis, and by increased consumption in DIC. Paternoster *et al*. reported that women with a HELLP syndrome had higher concentration of fibronectin and D-dimer and lower antithrombin levels than in normal pregnancy and in preeclampsia [85]. *Abruptio placentae *associated with the HELLP syndrome increases the risk of DIC substantially as well as the risk of pulmonary oedema, renal failure (oliguria, anuria, high serum creatinine levels) and the need for blood transfusion [9,35,86]. A contributing factor for acute renal failure is microangiopathy and DIC [4,59,60]. Visual disturbances, including retinal detachment, vitreal haemorrhage and cortical blindness, are infrequent complications in which DIC probably contributes [34].

### Maternal mortality

In a large retrospective cohort study comprising 442 pregnancies complicated by the HELLP syndrome, the maternal mortality was 1.1% [14], which is in accordance with other reports [3,9,87,88]. However, higher maternal mortality, up to 25%, has been reported [11]. Unexpected rapid death from HELLP may require forensic expertise [89]. Isler *et al*. found cerebral haemorrhage or stroke to be the primary cause of death in 26% and the most contributing factor in another 45% of the deaths [90]. Maternal mortality rate in hepatic rupture ranges from 18 to 86% [91].

### HELLP, perinatal mortality and morbidity

Perinatal mortality and morbidity are considerably higher in the HELLP syndrome than that of the mothers, and are primarily dependent on the gestational age when the condition develops [74,92]. The perinatal mortality rate related to the HELLP syndrome is between 7.4% and 34% [9,72,73]. Neonates delivered before completed 32 weeks' gestation have the highest risk of perinatal death [74,92]. According to Gul *et al*. the perinatal mortality was 34% before 32 weeks' gestation, and 8% after the 32nd gestational week [72]. Prematurity, placental insufficiency, with or without intrauterine growth restriction (IUGR) and *abruptio placentae*, are the leading causes of neonatal death [6,15,21]. Hepatic rupture has a perinatal mortality that can reach 80% [91].

Neonatal thrombocytopenia occurs in between 15% and 38% of cases [5,93] and is a significant risk factor for both intraventricular haemorrhage (IVH) and long-term neurological complications [5,94].

The neonatal outcome of the HELLP syndrome represents a controversy [94]. Some authors report that infants born to mothers with a HELLP syndrome are more likely to be small for gestational age (SGA) and to have increased risk of perinatal asphyxia and RDS [94] and that the respiratory and cardiovascular morbidity may be further aggravated by maternal HELLP occurring before 32 weeks' gestation [95]. A retrospective study was published in 2003 by Roelofsen *et al*. on the outcome of infants born after pregnancies complicated by HELLP or ELLP syndrome. The gestational age was 29.9 in the HELLP group and 30.3 weeks in the ELLP group. 64% were born before 32 weeks' gestation. Cerebral haemorrhage occurred in 3 of the infants in the HELLP group (all had thrombocytopenia (9–42·10^9^/L), none in the ELLP group. After 18 months four infants belonging to the HELLP group had major handicaps, none in the ELLP group, making a total adverse outcome of 22.8% [44].

Other authors inform that infants born to mothers with the HELLP syndrome are not at increased risk of morbidity compared to otherwise healthy infants of the same gestational age [92,93,96,97] and that that gestational age at delivery and birth weight primarily affected the perinatal mortality rather than the severity of the hypertensive disease [73]. Consequently also typical complications following preterm delivery in itself are reported, like bronchopulmonary dysplasia (BPD) cerebral haemorrhage and persisting *ductus arteriosus *in HELLP [44].

Murray *et al*. published in 2001 the outcome of 20 cases of the HELLP syndrome over a 5-year period. 85% were delivered by Caesarean section within 24 hours of diagnosis. 65% were preterm. The mean gestation at delivery was 33.5 weeks and the mean birth weight 1923 g. 40% of the neonates developed respiratory distress syndrome (RDS). The neonatal morbidity was most closely related to the gestation at delivery [77].

Analysis of the perinatal and neonatal data for women diagnosed with HELLP from 1993 to 1996 was performed by Singhal *et al*. who compared the neurodevelopmental outcome of HELLP a group of birth weight matched controls. A total of 109 infants (mean gestational age 32.6 weeks, mean birth weight 1766 g) were born by 104 women with HELLP syndrome. There were no significant differences in gender, Apgar score, need for resuscitation, RDS, sepsis, NEC or death in the neonatal unit, suggesting that infants born to mothers with a HELLP syndrome are not at increased risk for mortality or morbidity and that the majority of neonatal complications were attributable to prematurity [94]. There was a significant decrease in mortality and morbidity with increasing gestational age and birth weight. No significant differences in neonatal mortality and morbidity were found in infants weighing less than 1250 g compared to the weight matched control group. At 3 years of age, the HELLP group had fewer children with cerebral palsy (CP) and mental disability [94].

Kandler *et al*. reported that in the time span between 6 and 72 months (median 24 months) after delivery, 90% of children born from mothers with HELLP showed normal development or only minor disabilities. The mean gestational age was 33 weeks and the mean birth weight 1671 g [97]. However, the neonatal outcome is poor before 25 weeks' gestation or with birth weights less than 700 g; after 26 weeks' gestation or in infants weighing more than 700 g it is substantially better [74,92].

We assume that differences in the outcome of the neonates depend on the study publication and also reflect the level of neonatal care. Infants born from mothers with the HELLP syndrome may develop thrombocytopenia and associated CP. However, it seems that a low gestational age at delivery is the main problem rather then HELLP in itself. Most neonates born from a woman with HELLP have a normal long-term development.

### Management of pregnant women with HELLP syndrome

In general, there are three major options for the management of women with severe preeclampsia and HELLP syndrome [7,9,72,98]. These include:

1) Immediate delivery which is the primary choice at 34 weeks' gestation or later.

2) Delivery within 48 hours after evaluation, stabilization of the maternal clinical condition and CS treatment. At 27 to 34 weeks of gestation, this option appears appropriate and rational for the majority of cases.

3) Expectant (conservative) management for more than 48–72 hours may be considered in pregnant women before 27 weeks' gestation. In this situation, CS treatment is often used, but the regimens vary considerably.

### Conservative management (> 48 hours)

Large randomized clinical trials aimed to compare conservative versus aggressive management with immediate delivery of women with the HELLP syndrome are missing. However, expectant management before completed 34 weeks' gestation may be an acceptable option in selected cases if it is performed in tertiary care units under close maternal and foetal surveillance (e.g. antihypertensive treatment, ultrasound and Doppler examination) [99,100]. Possible advantages due to limited prolongation of pregnancy should be carefully weighed against the increased risks for maternal and foetal complications (*abruptio placentae*, acute renal failure, pulmonary oedema, DIC, perinatal and maternal death) [10]. If the maternal condition worsens, immediate Caesarean section is inevitable [99,100]. Conservative treatment is contraindicated in women with DIC [58].

The benefit of temporizing management of HELLP syndrome is questioned [10,101]; some authors warn against expectant management to optimize maternal condition before delivery beyond 24–48 hours [10] or conservative management is disregarded [73]. However, expectant management of pregnant women with HELLP syndrome remote from term is common practice in the Netherlands, conditional on the safety of the mother [20,102].

### Corticosteroid (CS) treatment

#### Promotion of foetal lung maturation in threatening preterm delivery

Irrespective of the underlying condition, preterm delivery (< 37 weeks' gestation) carries the risk of RDS in neonates because of insufficient surfactant production in foetal lungs. The neonates can be treated with CS and surfactant. Prenatal CS treatment has been shown to accelerate foetal lung maturation through a complex interaction of hormonal and intercellular signalling that leads to differentiation of the surfactant lipid-protein pathway and through less well-defined increases in lung compliance [103]. The foetal lung must be biologically ready for a CS to "trigger" maturation. In humans this window of biological readiness of the lungs seems to occur most often between 26 and 33 weeks' gestation [103].

Recently, betamethasone, instead of dexamethasone, has been recommended as a drug of choice for promotion of foetal lung maturation in threatening preterm delivery [104]. In clinical trials as well as observational studies antenatal CS treatment is associated with a decreased risk of IVH and CP [105]. Betamethasone may be safer and more protective of the immature brain than dexamethasone [106].

In a retrospective cohort study by Baud *et al*. comprising 883 infants with gestational age between 24 and 31 weeks it was reported an odds ratio (OR) for cystic periventricular leucomalacia of 0.5 (95% confidence interval (CI) 0.3–0.9) for betamethasone compared with no treatment and 1.5 (95% CI 0.8–2.9) for the dexamethasone treated group [107]. Treatment of severe preeclampsia with betamethasone in the time span between 26 and 34 weeks' gestation has been shown to significantly reduce the rate of RDS, IVH and perinatal death in preterm delivery [108]. A Cochrane update from 2006 advocated a single course of antenatal CS (12 mg betamethasone twice) in gestational ages between the 26th and 35th gestational weeks [109]. Thus, a single course of CS is advocated in threatening preterm delivery, including severe preeclampsia.

#### Multiple courses are more effective, but may harm the foetus

Two randomized trials of women at risk of preterm delivery showed that weekly CS caused less RDS, less severe neonatal lung disease and serious neonatal morbidity and less need for mechanical respiratory support and surfactant use [110,111]. The short-term benefits supported the use of repeated doses of CS in women who remain at risk of very preterm birth 7 or more days after an initial course. However, both studies raised serious concerns about lower neonatal birth weight in the repeat course group [110,111]. Two long-term follow-up studies have been presented [112,113]. One favours the use of repeated CS courses [112]. In the other a non-significant higher rate of CP was detected in the repeat group (6 versus 1) and it was concluded that present findings indicate no evident long-term benefit, rather a possible harm, arguing that weekly administration of antenatal CS should not be offered after an initial course [113]. Repeated CS exposure may increase mortality, restrict foetal growth and cause prolonged foetal adrenal suppression [114,115]. Both repeat maternal CS exposure and early neonatal dexamethasone treatment may cause CP in preterm neonates [113,116,117]. An increase in the prevalence of CP among very preterm deliveries (time span 24 to 30 weeks of gestational age) has been reported. Postnatal dexamethasone treatment was associated with higher rates of CP, whereas antenatal CS treatment was associated with lower rates [117]. Early dexamethasone treatment should not be recommended for the routine prevention or treatment of chronic lung disease [118].

#### CS treatment for the women with HELLP syndrome

Whereas delivery is the mainstay of treatment for the HELLP syndrome, CS treatment is a possible addendum. Present alternatives for CS treatment are:

1) standard CS treatment to promote foetal lung maturity

2) high-dose dexamethasone treatment of the mother

or

3) treatment with repeated doses to reduce maternal morbidity and hastening recovery.

Maternal benefit of CS treatment for the HELLP syndrome was first reported in 1984 [119]. In addition to accelerate maturation of the foetal lungs, favourable maternal effects of CS treatment have been suggested: diminished oedema, inhibited endothelial activation and reduced endothelial dysfunction, prevention of thrombotic microangiopathic anaemia, and inhibition of cytokine production and thereby induce anti-inflammatory effects in the HELLP syndrome [27]. Benefit from CS treatment of the HELLP syndrome was reported in a publication from 1993 where less frequent grade III and IV IVH, necrotizing enterocolitis (NEC), retrolental fibroplasia and fewer neonatal deaths were observed [120]. In addition to accelerate foetal lung maturity, antenatal CS has been used to reduce the risk of IVH and NEC in selected cases of the HELLP syndrome with maternal PLT count over 50·10^9^/L between 24 and 34 weeks' gestation during continuous maternal and foetal surveillance [121].

Thus, CS treatment of the mother with HELLP looks theoretically attractive.

#### Evaluation of standard CS treatment on maternal HELLP

It remains uncertain if standard CS treatment to induce foetal lung maturation has been convincingly shown to benefit the woman with HELLP [9]. A Cochrane analysis from 2004 concluded that CS treatment did not affect maternal mortality and outcomes such as placental abruption, pulmonary oedema and liver complications. There was a shorter mean duration of hospital stay (4.5 days in favour of corticosteroids over placebo) and a tendency toward greater PLT count increase over 48 hours [122]. A recent review confirmed that CS increased the PLT count without improving maternal morbidity in the HELLP syndrome [37]. Thus, standard CS treatment has only minor clinical effects in the HELLP syndrome. Strong evidence for recommending standard CS treatment in women with the HELLP syndrome has not been presented.

#### High-dose dexamethasone treatment of maternal HELLP

Retrospective and small randomized studies suggested that the use of high-dose dexamethasone (10 mg dexamethasone every12 hours) in the HELLP syndrome reduced maternal morbidity and induced more rapid improvement of the PLT counts. Thereby the rate of regional anaesthesia could be increased, consequently allowing vaginal delivery [27,87,123-128]. In a publication from 2006 by Martin *et al*. (based on retrospective analysis and referring to experience and publications and 2 small randomised studies in the antepartum period that reported less morbidity in the treatment group), aggressive use of potent GS was recommended as a cornerstone of management for women with the HELLP syndrome class 1 and 2 or for women with class 3 HELLP syndrome accompanied with epigastric pain, eclampsia, severe hypertension or evidence of major organ morbidity [7]. CS treatment was recommended only as short-term intervention. Continuation of pregnancy for more than 48 hours after CS administration for very preterm HELLP syndrome can lead to significant maternal and foetal morbidity and mortality [7,129].

The largest randomized double blind, placebo controlled (dexamethasone versus placebo) study so far by Fonseca *et al*. included 132 women with the HELLP syndrome. The study included both HELLP occurring in pregnancy (n = 60) and after delivery (n = 72) [130]. This study could not confirm favorable results of previous small studies. Dexamethasone treatment did not reduce maternal complications (such as acute renal failure, pulmonary oedema and oliguria). The rates of platelets and fresh frozen plasma transfusions were not significantly reduced, nor was the time of recovery of laboratory test shortened, or the duration of hospital stay. The results of this study did not support the routine use of high-dose dexamethasone [130].

#### Summary and general comments on CS therapy in the HELLP syndrome

In threatening preterm delivery a single course of CS has documented clinical benefit for the foetus without adverse effects. Multiple courses should be avoided, except well-structured research protocols [131]. Although CS treatment has been shown to be effective in severe preeclampsia, it seems to be less beneficial in the HELLP syndrome [9]. The largest randomized trial on high-dose dexamethasone did not support high-dose dexamethasone treatment of the mother with the HELLP syndrome. Its routine use is disregarded by Sibai who advocates standard-dose CS treatment (either 2 doses of betamethasone every 12 hours intramuscularly, or 6 mg dexamethasone intravenously every 12 hour) to improve perinatal outcome in the HELLP syndrome diagnosed between 24 and 34 weeks' gestation and then deliver 24 hours after the last dose of CS [9].

In a recent review, Vidaeff and Yeomas pointed out that available evidence does not support that CS treatment can improve the outcome of pregnancies affected by the HELLP syndrome either antepartum and/or post-partum. Benefits from CS treatment for disease modification in the HELLP syndrome should individually be compared with immediate delivery, the current gold standard [132]. Thus, there is strong evidence for a single course of standard CS treatment in preterm delivery, including severe preeclampsia, but no conclusive evidence supporting CS treatment of the HELLP syndrome.

#### Practical approach towards a woman with a suspect or diagnosed HELLP syndrome

The first step is to evaluate the patient. The clinical maternal status, gestational age (ultrasound determined), presence of labour and cervical Bishop score should be determined. The laboratory examination must include complete blood cell count, in particular PLT count, coagulation parameters, AST, LDH and haptoglobin and urine examination. Blood pressure measurement, ultrasound examination and foetal assessment tests (Cardiotocography and Doppler examination) are important [5]. The next step is to stabilize the maternal clinical condition with intravenous fluids, antihypertensive drugs (e.g. labetalol or nifedipine) and magnesium sulphate to prevent convulsions [5,7,9,15]. It is of utmost importance to monitor closely maternal vital signs and fluid balance [5]. We agree with the proposal by Sibai [9] and others [7,133] who do not generally recommend immediate Caesarean section, but advocate vaginal or Caesarean delivery 24 to 48 hours after CS treatment for maximum maternal and foetal benefits. However, according to recent literature there is no strong evidence for beneficial effects following CS treatment in the HELLP syndrome. If the HELLP syndrome develops before 24 weeks' gestation, termination of pregnancy should be strongly considered [134].

#### Other treatment options

Treatment with antithrombin has been suggested as a possible therapeutic option for preeclampsia [135]. In a randomized study of severe preeclampsia it was shown that antithrombin supplementation may correct hypercoagulability, stimulate prostacyclin production, regulate thrombin-induced vasoconstriction, improve foetal status (improving foetal biophysical profile, decreasing foetal distress) and promote foetal growth [136]. In contrast to the use of heparin, antithrombin has not been shown to increase the risk of bleeding. However, antithrombin treatment has so far not been investigated in randomized studies in women with the HELLP syndrome. The potential benefit from antithrombin treatment of women with HELLP syndrome might be a reasonable objective to be tested in future well designed multicenter studies.

It is well documented that glutathione levels are decreased in the HELLP syndrome. Enhancement of intracellular glutathione may protect against damage by hydrogen peroxide. Normalization of the glutathione levels in patients with preeclampsia and the HELLP syndrome might be a promising therapy in the future [137]. Moreover, infusion of S-nitrosoglutathione in women with severe preeclampsia lowers maternal mean arterial pressure, reduces platelet activation and uterine artery resistance without further compromising foetal Doppler indices [138]. Ruptured subcapsular liver haematomas must lead to Caesarean section. Operative treatment is not usually required if unruptured [38]. Spontaneous rupture of a subcapsular liver haematoma may be treated by surgery (packing with gauze is preferable to lobectomy), arterial ligation or selective arterial embolization or even liver transplantation [61-63,65,78]. Recombinant factor VIIa is an effective adjunct in the treatment of preeclamptic patients with expanding or ruptured subcapsular liver haematoma [64]. Pulmonary oedema may be more effectively treated by applying early haemodialysis [139]. It is interesting that *in utero *exposure of magnesium sulphate may be protective against development of CP [140,141]. Its neuroprotective effect has been shown in neonatal animals with experimental brain lesions [94].

#### Timing and mode of delivery

We have not identified any randomized trial comparing maternal and neonatal outcome after vaginal delivery or Caesarean section for women with the HELLP syndrome. The indication of delivery, timing and method of delivery of the HELLP syndrome is more or less depending on experience and local traditions and there is no general agreement. A woman with a class 3 HELLP syndrome could await spontaneous onset of delivery at term [15]. Pregnant women with moderate (class 2), complete or severe (class 1) HELLP syndrome with completed 34 weeks' gestation should be delivered immediately after control of maternal hypertension [15]. The route of delivery should be selected on obstetric indications including cervical status, obstetric history, the maternal and the foetal condition. If the cervix is unfavourable for induction of labour, cervical ripening should be the first step [21].

Before 34 weeks' gestation, delivery should be chosen if the maternal condition cannot be controlled rapidly, if the maternal condition worsens or signs of intrauterine foetal distress develop. Maternal indications for immediate delivery include blood pressure > 160/110 mmHg despite treatment with antihypertensive drugs, persisting or worsening clinical symptoms, deteriorating renal function, severe ascites, *abruptio placentae*, oliguria, pulmonary oedema or eclampsia [72]. In such cases most clinicians probably will prefer Caesarean section.

In pregnant women between 24 and 34 weeks' gestation a full course of CS is advocated after maternal stabilization (particularly blood pressure and coagulation abnormalities) followed by induced delivery after 24 hours [7,9]. However, as mentioned, the support for this regimen is weak. Caesarean section should be performed in women who develop HELLP syndrome before 30 weeks' gestation [9,142] and in those in whom oligohydramnion and/or unfavourable Bishop score are diagnosed [9]. Regional anaesthesia is indicated for cases with PLT counts below 100·10^9^/L. However, epidural anaesthesia is contraindicated if the PLT count is below 75·10^9^/L [9]. Some authors also claim that regional anaesthesia is contraindicated if the platelet count is below 100·10^9^/L [143]. Platelet transfusion prior to Caesarean section has been suggested for class 1 HELLP syndrome, and for those with vaginal delivery and PLT count below 20 to 25·10^9^/L [21]. Antihypertensive drug is administered to keep blood pressure below 155/105 mmHg, and the woman should be monitored closely for at least 48 hours after delivery [10]. Most patients show evidence of resolution during this time.

#### Management of post-partum HELLP syndrome

In most women with a HELLP syndrome, the maternal PLT counts continue to decrease immediately post-partum with an increasing trend on the third day [6]. About 30% of the HELLP syndromes develop after birth; the majority within the first 48 hours. However, the time of onset might range from a few hours to 7 days after delivery [10]. In women with post-partum HELLP syndrome, risk of renal failure and pulmonary oedema is significantly increased compared to those with an antenatal onset [59,86]. Since early post-partum administration of high-dose CS might accelerate recovery [128], its routine administration is highly advocated (10 mg of dexamethasone every 12 hours) [6,144-146].

However, a randomized study showed that adjunctive use of intravenous dexamethasone for postpartum patients with severe preeclampsia did not reduce disease severity or duration [147]. Moreover, the benefit of dexamethasone in post-partum HELLP syndrome was not verified in a randomized placebo controlled trial on 105 women with postpartum HELLP syndrome. There was no difference in maternal morbidity, duration of hospital stay, need for rescue scheme or the use of blood products between the groups, nor was there any difference with respect to the pattern of PLT count, recovery, AST, LDH, haemoglobin or diuresis. These findings did not support the use of dexamethasone in the puerperium for recovery of women with HELLP [148].

Women with a HELLP syndrome who demonstrate progressive elevation of bilirubin or creatinine for more than 72 hours after delivery may benefit from plasma exchange with fresh frozen plasma [149-151]. In the case of continuing haemolysis, persistent thrombocytopenia and hypoproteinaemia, post-partum erythrocyte and thrombocyte substitution, as well as albumin supplementation, are standard treatment regimens [5,21]. In a recent study of women with class 1 HELLP syndrome adding of platelet transfusion to standard CS administration did not increase the recovery rate [152]. Ertan *et al*. treated women with diuresis problems in the postpartum period with furosemide and applied prophylaxis with antithrombin or low-dose heparin for DIC [5]. A meta-analysis concluded that furosemide was not beneficial to prevent or treat acute renal failure in adults [153]. Too little fluid can exacerbate an already vasoconstricted intravascular volume and lead to renal injury in severe preeclampsia or in the HELLP syndrome. A bolus intravenous fluid of 250–500 ml is advocated if oliguria persists, and, if necessary, central monitoring of the patient [6].

Some patients with the HELLP syndrome, especially those with DIC, may demonstrate delayed resolution or even deterioration in the post-partum period [101]. Therefore, the use of heparin has been proposed for patients with preeclampsia, HELLP syndrome and DIC. A retrospective analysis of women with DIC in the post-partum period revealed that 6 of 9 developed post-partum bleeding including retroperitoneal haematoma. Treatment with heparin was discouraged for post-partum bleeding [84]. Thus, most authors oppose the routine use of heparin.

#### Risk of recurrence and pre-conceptional counselling

Sibai has shown that oral contraceptives are safe in women with a prior HELLP syndrome [8]. Women with a history of the HELLP syndrome carry an increased risk of at least 20% (range 5–52%) that some form of gestational hypertension will recur in a subsequent gestation [7,11,101,142,154].

In a subsequent pregnancy, women with a history of HELLP syndrome at or before 28 weeks' gestation during the index pregnancy are at increased risk for several obstetric complications (preterm birth, pregnancy-induced hypertension and increased neonatal mortality) [154]. In patients with prior severe, early-onset preeclampsia thrombophilia screening has been suggested and should include search for protein S deficiency, activated protein C resistance (APC resistance), hyperhomocysteinemia and anti-phospholipid antibodies (both lupus anticoagulant (LA) and anti-cardiolipin) [155].

### Concluding remarks

Different definitions and classifications have up to now been used to diagnose the HELLP syndrome. This has limited the usefulness of many clinical reports. The Tennessee and Mississippi classifications are well suited to facilitate comparisons. Classifications used in future reports should be restricted to one of these.

Neither randomized trials nor Cochrane evaluations regarding delivery in women with the HELLP syndrome have been performed. In order to reduce the risk of potentially serious complications, there is consensus that early delivery is indicated when the HELLP syndrome develops after 34 weeks of pregnancy. Expectant management and the use of CS in the HELLP syndrome developed prior to 34 weeks of gestation are main controversial issues. There is no general agreement on timing of delivery and the best method of delivery. In deliveries in the time-span between 24 and 34 weeks' gestation, a standard CS course is usually recommended after stabilization of the maternal condition, followed by delivery 24 hours later. Although a maternal benefit has been demonstrated for patients with severe preeclampsia, this effect seems to be limited or lacking in patients with the HELLP syndrome. Repeated CS dosage and high-dose dexamethasone treatment can at present not be recommended. There is a definite need both in antepartum and post-partum HELLP patients for adequately sized, randomized, placebo-controlled trials regarding dosage of CS, as well as high-dose dexamethasone versus standard CS dosage. Better insight in the complex pathophysiology of the HELLP syndrome may lead to new treatment alternatives and improved clinical management. A well designed multicenter study testing the benefit of antithrombin to counteract DIC in the HELLP syndrome should be encouraged.

## Abbreviations

α-GST: glutathione S-transferase; AFLP: acute fatty liver of pregnancy; ALAT: alanine aminotransferase; APS: antiphospholipid syndrome; AST: asparate aminotransferase; CI: 95% confidence interval; CS: corticosteroid; CP: cerebral palsy; CT: computerized tomography; DIC: disseminated intravascular coagulation; ELLP: elevated liver enzymes, low platlets; FDP: fibrin degradation products; GT: gestational thrombocytopenia; HELLP: H = Haemolysis, EL = Elevated Liver enzymes, LP = Low Platelets; HUS: haemolytic uremic syndrome; IUGR: intrauterine growth restriction; ITP: immune thrombocytopenic purpura; IVH: intraventricular, haemorrhage; LA: lupus anticoagulant; LDH: lactate dehydrogenase; MAHA: microangiopathic haemolytic anaemia; MRI: magnetic resonance imaging; NEC: necrotizing enterocolitis; OR: odds ratio; PLT: platelet; PT: prothrombin time; PTT: partial thromboplastin time; RDS: respiratory distress syndrome; SGOT: glutamic oxaloacetic transaminase; SGPT: serum glutamic transaminase; SLE: systemic lupus erythematosus; TTP: thrombotic thrombocytopenic purpura; U/L: units/L.

## Competing interests

The authors declare that they have no competing interests.

## Authors' contributions

KH has provided most part of the literature search and also written all initial chapters in the publication and performed most part of the revision. ES has contributed concerning pathology issues, provided literature and participated from the beginning in the writing process. UA has contributed regarding medical issue, differential diagnosis, complications, and in the writing process. All authors have read and approved the final version.

## Pre-publication history

The pre-publication history for this paper can be accessed here:


